# Attitudes and Preferences Toward a Hypothetical Trial of an Internet-Administered Psychological Intervention for Parents of Children Treated for Cancer: Web-Based Survey

**DOI:** 10.2196/10085

**Published:** 2018-12-18

**Authors:** Joanne Woodford, Anna Wikman, Kim Einhorn, Martin Cernvall, Helena Grönqvist, Amanda Romppala, Louise von Essen

**Affiliations:** 1 Clinical Psychology in Healthcare Department of Women's and Children's Health Uppsala University Uppsala Sweden

**Keywords:** anxiety, cancer, clinical trial, depression, eHealth, parents

## Abstract

**Background:**

Clinical trials are often challenged with issues of recruitment and retention. Little is known concerning general attitudes and preferences toward trial design and willingness to participate among parents of children treated for cancer. Furthermore, willingness to participate in internet-administered psychological interventions remains unexplored. In this study, we examined attitudes and preferences of the population regarding study procedures for a hypothetical trial of an internet-administered psychological intervention. In addition, differences in the response rate between modes of study invitation and willingness to engage in internet-administered interventions were examined.

**Objective:**

The primary objective of this study was to examine attitudes and preferences toward participating in an internet-administrated psychological intervention. The secondary objective was to examine the response rates and help-seeking behavior among parents of children treated for cancer.

**Methods:**

A cross-sectional, Web-based survey was conducted with parents of children who had completed cancer treatment. This Web-based survey examined self-reported emotional distress, prior help-seeking and receipt of psychological support, past research participation, attitudes toward research, preferences concerning recruitment procedures, and attitudes toward different types of trial design.

**Results:**

Of all the parents invited, 32.0% (112/350) completed the survey, with no difference in response rate between modes of study invitation (χ^2^_1_=0.6, *P*=.45). The majority (80/112, 71.4%) of parents responded that they had experienced past emotional distress. Responses indicated high (56/112, 50.0%) or somewhat high trust in research (51/112, 45.5%), and the majority of parents would accept, or maybe accept, internet-administered psychological support if offered (83/112, 74.1%). In addition, responses showed a preference for postal study invitation letters (86/112, 76.8%), sent by a researcher (84/112, 75.0%) with additional study information provided on the Web via text (81/112, 72.3%) and video (66/112, 58.9%). Overall, parents responded that trials utilizing a waiting list control, active alternative treatment control, or a patient-preference design were acceptable.

**Conclusions:**

Parents of children treated for cancer appear willing to participate in trials examining internet-administered psychological support. Findings of this study will inform the design of a feasibility trial examining internet-administered psychological support for the population.

## Introduction

### Background

Due to marked treatment advances across the developed world, the overall 5-year survival rate of children diagnosed with cancer is now >80% [1]. However, a number of negative impacts of living with childhood cancer persist long term, for both parents [2] and childhood cancer survivors [3,4]. In the case of parents, negative impacts include financial difficulties [5,6], uncertainty regarding future prognosis [7], and poor quality of life [8]. Furthermore, a considerable proportion of parents report elevated levels of long-term psychological distress [9-11]. However, despite increased psychological distress, parents have reported substantial unmet health care needs concerning the receipt of psychological support in Sweden [12] and Australia [13,14]. Potential reasons for these unmet psychological needs include the high costs of delivering psychological treatment, alongside a lack of qualified therapists [15]. Indeed, research indicates psychological support is not routinely offered to parents in Sweden [11,12]. Furthermore, parents may experience additional barriers to accessing psychological support such as lack of time, guilt, and putting their child’s health first [16].

Electronic mental health interventions (eMental Health) may represent a way of increasing access to psychological support [17] for parents of childhood cancer survivors. One example of an eMental Health intervention is internet-administered cognitive behavioral therapy (ICBT) [18]. ICBT is a clinically and cost-effective psychological intervention for a range of mental health difficulties [19] and has been demonstrated to be as effective as face-to-face psychological interventions [20]. Furthermore, ICBT may overcome barriers to accessing psychological support, such as parental guilt, considering its increased anonymity in comparison with face-to-face treatment [21]. In addition, as treatment provision is not confined to specific locations or times, practical barriers (eg, lack of time) may be overcome [21]. Given the potential of ICBT as a solution, a research program, informed by the Medical Research Council (MRC) framework for complex interventions [22], has been undertaken to develop an ICBT intervention tailored to the needs of parents of children treated for cancer [10,23].

This study builds upon the previous MRC phase I development [24] work [10,23] by recognizing the necessity to examine the feasibility of methodological and study procedures before conducting definitive controlled trials [25]. Successful recruitment, retention, and data completeness are essential for clinical trials to reach power and maximize generalizability [26]. However, recruitment difficulties in psychological treatment trials are common [27], including ICBT trials [28]. Indeed, in our previous research, we encountered difficulties with recruitment and attrition into a randomized controlled trial (RCT) examining an ICBT intervention for parents of children currently receiving treatment for cancer [29,30]. While trial design can contribute to poor recruitment [31] and attrition [32], understanding attitudes and preferences toward trial design may improve acceptability and, subsequently, recruitment and retention [33]. However, the existing literature is scarce on attitudes and preferences toward ICBT trial design, as well as willingness to participate among parents of children treated for cancer. As such, a survey study was conducted to examine attitudes and preferences regarding the design of a hypothetical trial of an internet-administered psychological intervention, alongside study response rates, past and present emotional distress, and help-seeking behavior. Results will be used to inform the design of a planned phase II feasibility trial (MRC) of the ICBT intervention previously developed for parents of children treated for cancer [10,23].

### Aims and Objectives

The primary study aims were to examine (1) attitudes and preferences toward trial design and (2) willingness to participate in a hypothetical trial of internet-administered psychological intervention for parents of children treated for cancer. Secondary study aims were to investigate (1) overall response rates and differences in the response rate between two modes of study invitation and (2) current and past emotional distress and help-seeking behavior. The following outcomes were examined:

1. Overall response rate
2. Number of study invitation reminders required to recruit
3. Differences in the response rate between two modes of study invitation (standard letter and professionally illustrated postcard)
4. Self-reported current and past emotional distress
5. Prior help-seeking and receipt of psychological support
6. Willingness to receive internet-administered psychological support
7. Past experience of research participation and attitudes toward research
8. Attitudes and preferences concerning the delivery of initial study invitations and presentation of full study information
9. Attitudes toward participation in different trial designs (waiting list control, alternative active treatment, and patient-preference).

## Methods

### Study Design

A cross-sectional, Web-based, self-report survey was conducted from April 2017 to June 2017 with parents of children treated for cancer and recruited across Sweden.

### Eligibility Criteria

In this survey study, Swedish-speaking parents of children treated for cancer (aged 0-16 years at study commencement) residing in Sweden were eligible. Parents were included if the child had (1) completed successful cancer treatment 3 months to 5 years earlier at study commencement and (2) been treated in 1 of the 6 pediatric oncology units in Sweden. Parents were excluded if the child had a benign tumor.

### Recruitment and Study Procedures

Potential participants were identified using a two-step screening process:

1. Childhood cancer survivors, meeting the inclusion criteria, were identified via the Swedish Childhood Cancer Registry (National Quality Registry, initiated in 1982).
2. Children’s personal identification numbers were linked to parents’ personal identification numbers via the SPAR-Registry (“Statens personadressregister” by Swedish acronym) held by the Swedish Tax Agency to obtain parent contact information.

Although the SPAR-Registry includes all currently registered residents in Sweden, at the time of the study, it was only possible to access parent information for children aged ≤16 years. Therefore, the current age-span of children, whereby it was possible to identify parents, was restricted to 0-16 years.

From identified parents, an in-house computer program was used to randomize to the mode of invitation (letter vs postcard), with a 1:1 allocation, stratified by cohabitation status (cohabiting parents, noncohabiting parents, one parent registered). Prior to posting invitations, the most up-to-date information concerning whether children were currently living, or deceased, was checked via the telephone by a member of the research group with the Swedish Tax Agency.

**Figure 1:**
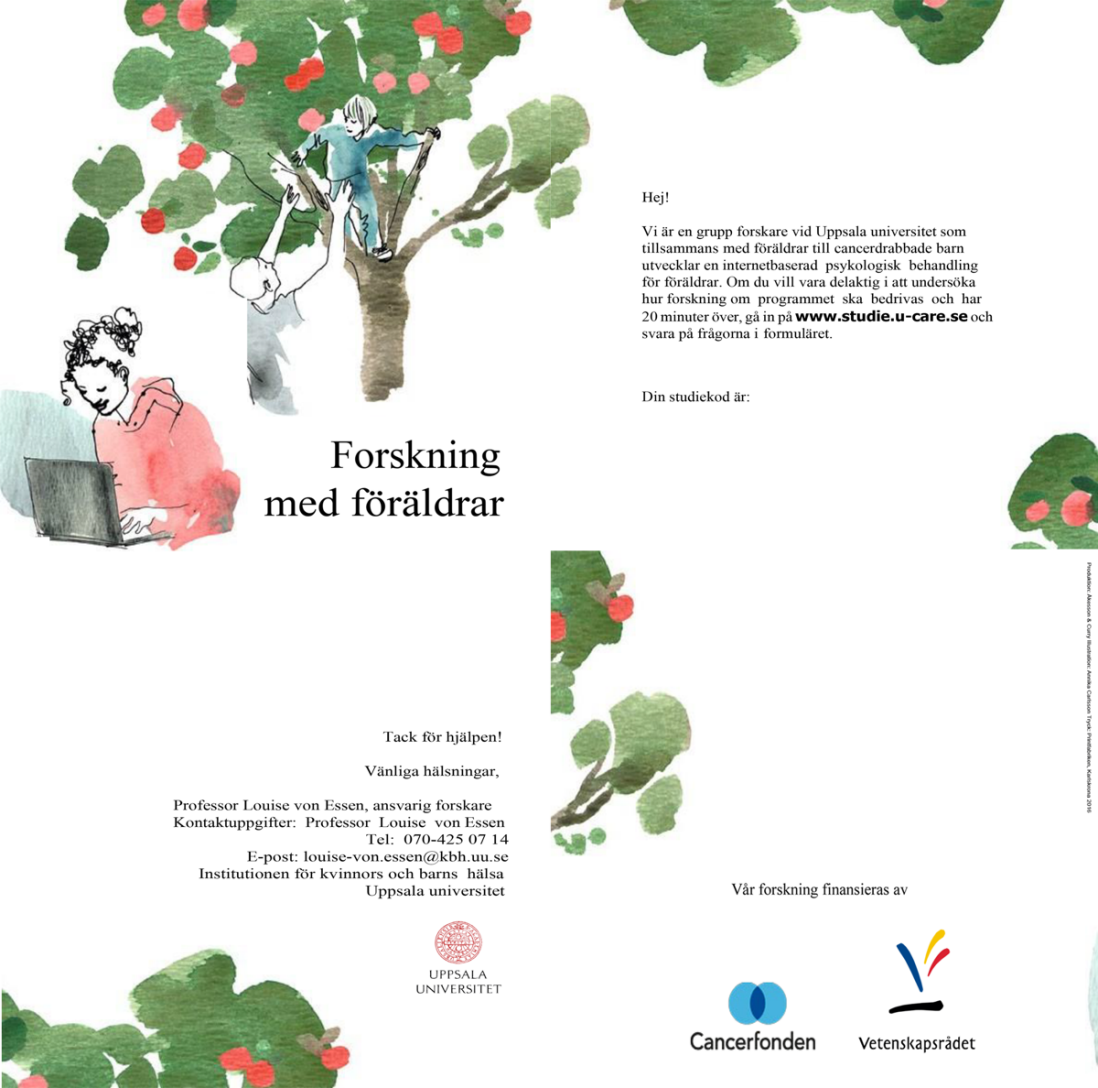
Study invitation postcard.

**Figure 2:**
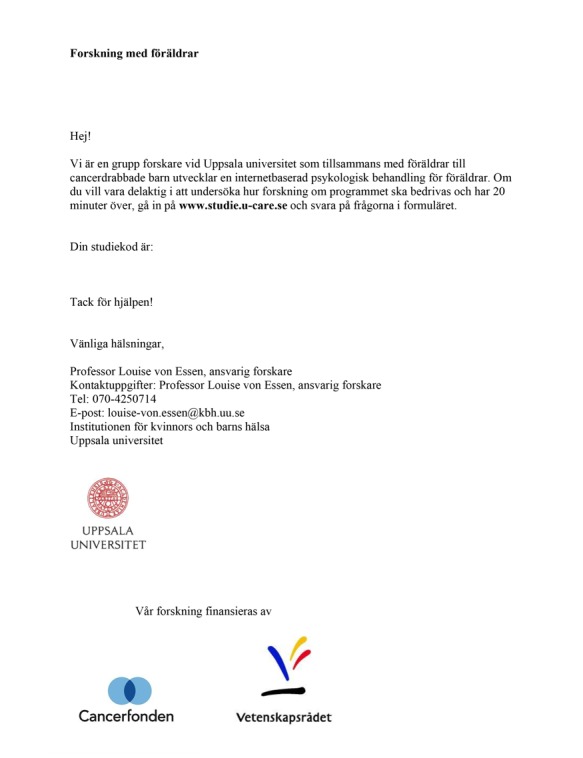
Study invitation letter.

Parents were sent an invitation either via a postcard (Figure 1) or a letter (Figure 2). Each mode of invitation contained identical text (see Multimedia Appendix 1 for the English translation), providing brief study information, a link to a Web portal (the U-CARE-portal), and an individual log-in code. The study invitation letter followed a standard plain letter format and was sent in the post using an envelope. The study invitation postcard was designed by a professional illustrator, including color illustrations representing the intervention and population. Invitation letters or postcards, with a “post-stick” reminder note, were resent at 1 week and 2 weeks postinitial study invitation to parents who did not respond.

Parents were provided full study information via the U-CARE-portal (Multimedia Appendix 2), and informed consent was provided through Web (Multimedia Appendix 3). After the provision of Web-based consent, parents gained access to the survey. Prior to completing the survey, parents were able to view a brief informational video (5 minutes and 23 seconds) presenting the background of the study. The brief informational video covered the following topics: (1) psychological distress in parents of children treated for cancer; (2) what is internet-administered psychological support; and (3) general information on clinical trials (eg, randomization and different types of control condition). Multimedia Appendix 4 provides the full video, and Multimedia Appendices 5 and 6 provide an English transcript of the video and translation of PowerPoint used in the video, respectively.

Ethical approval was obtained from the Regional Ethical Review Board in Uppsala (DNR: 2015/426/3).

### Sample Size

Sample size calculation indicated that a minimum of 340 participants would be required to detect a difference of 15% in the response rate between groups (ie, those who responded vs those who did not respond), with a power of 0.8 and *P*<.05 (two-tailed).

### Measures

#### Sociodemographics

The following sociodemographic data were collected for parents via the SPAR-Registry: (1) date of birth; (2) gender; and (3) marital status. Sociodemographic data for children were collected via the Swedish Childhood Cancer Registry: (1) cancer diagnosis; (2) date of diagnosis; (3) date of treatment completion; (4) date of birth; and (5) gender.

#### Web-Based Survey

A Web-based survey, consisting of 20 items and written in Swedish, was designed for the study and comprised 4 subsections as follows: (1) sociodemographics (3 items); (2) emotional distress and psychological support (6 items); (3) experience of participation in research and attitudes toward research (2 items); and (4) attitudes toward proposed trial procedures (9 items). The survey was administered on the U-CARE-portal, an internet research platform, designed to support both data collection and the provision of complex eMental Health interventions [34]. Multimedia Appendix 7 provides an English translation of the survey.

### Statistical Analysis

Study recruitment and flow are reported using an adapted version of the Consolidated Standards of Reporting Trials Statement [35]. Descriptive statistics were used to describe the study sample and compare those who responded and those who did not respond regarding key sociodemographic and clinical variables for parents and children. Descriptive data are reported in terms of numbers (*n*) and percentages (%) or means and SDs. Chi-square tests were used to assess differences between those who responded and those who did not concerning categorical data, and independent-sample *t* tests were used to assess differences regarding continuous variables (*P*<.05). Furthermore, chi-square tests were used to assess differences in response rate between parents invited via postcard and those invited via study invitation letter. Descriptive statistics are used to present the responses to the Web-based survey. All data analyses were performed using SPSS statistics for Windows, version 24.8.

## Results

### Study Recruitment and Participant Flow

**Figure 3:**
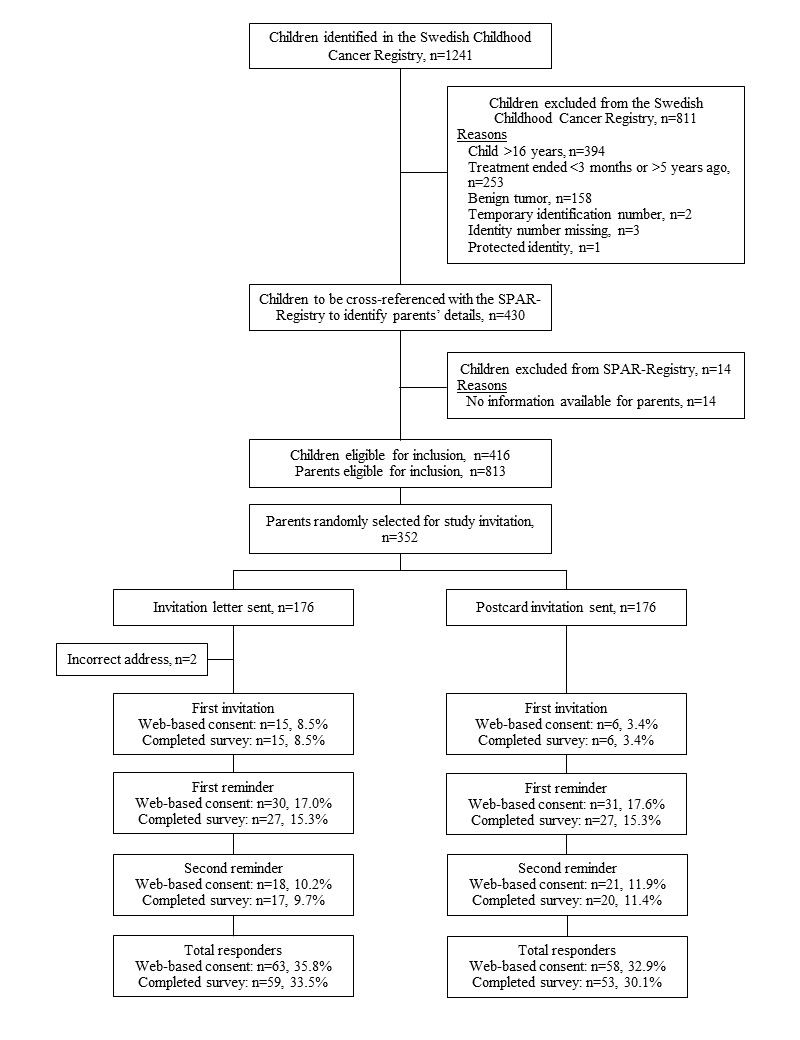
Study recruitment and flow.

Figure 3 presents study recruitment and flow. Of 1241 children identified in the Swedish Childhood Cancer Registry, 430 met the eligibility criteria. The parents’ contact information could be identified for 416 of these children via the SPAR-Registry, totaling 813 parents eligible for inclusion. A stratified random sample (n=352) was drawn for randomization to the mode of invitation proportional to the number of cohabitating, noncohabitating, and single parents among potentially eligible parents identified. The distribution of the sample was as follows: 81.8% (288/352) cohabitating parents; 15.9% (56/352) noncohabitating parents; and 2.3% (8/352) single parents (status of other parent unknown) listed on the SPAR-Registry. Due to incorrect contact information listed in the SPAR-Registry, 2 eligible parents did not receive the study invitation and were excluded. Of the invited parents (174 via study invitation letter and 176 via study invitation postcard), 34.6% (121/350) provided Web-based consent and 32.0% (112/350) completed the Web-based survey. Before completing the Web-based survey, 45.5% (51/112) parents watched over 3 minutes, 26.8% (30/112) watched 2-3 minutes, and 27.7% (31/112) watched less than 2 minutes of the informational video.

### Sociodemographic and Clinical Variables

The majority of parents who responded were female, cohabiting, with a mean age of 43.2 years. Children were predominantly male, had experienced leukemia, with a mean current age of 9.3 years, and had finished cancer treatment an average of 2.9 years ago. No significant differences were noted regarding parent or child sociodemographic and clinical variables between those who responded and those who did not (Table 1).

**Table 1:** Baseline characteristics of those who responded versus those who did not respond (N=350).

Characteristics			Parents who responded (n=112)	Parents who did not respond (n=238)		*P* value
**Parents**						
	**Gender, n (%)**				.13	
		Female	63 (56.3)	113 (47.5)		
		Male	49 (43.7)	125 (52.5)		
	**Marital status, n (%)**				.75	
		Cohabiting	90 (80.4)	196 (82.4)		
		Living apart	20 (17.8)	36 (15.1)		
		Unknown	2 (1.8)	6 (2.5)		
	Age in years, mean (SD)		43.2 (7.3)	43.1 (6.6)		.87
**Child**						
	**Gender, n (%)**				.73	
		Female	51 (45.5)	113 (47.5)		
		Male	61 (54.5)	125 (52.5)		
	**Cancer diagnosis category, n (%)**				.55	
		Leukemia	76 (67.9)	156 (65.5)		
		Central nervous system tumor	22 (19.6)	58 (24.4)		
		Solid tumor	14 (12.5)	24 (10.1)		
	Age in years, mean (SD)		9.3 (2.8)	10.0 (3.0)		.63
	Years since end of treatment, mean (SD)		2.9 (1.4)	2.9 (1.4)		.43

### Mode of Study Invitation and Number of Reminders

No difference was noted between the mode of invitation and response rate (letter: 59/112, 52.7%, vs postcard: 53/112, 47.3%; (χ^2^_1_=0.6, *P*=.45). Among those who responded, 18.8% (21/112) responded to the first invitation (letter: 15/21, 71.4%; postcard: 6/21, 28.5%), 48.2% (54/112) responded following one reminder (letter: 27/54, 50.0%; postcard: 27/54, 50.0%), and 33.0% (37/112) responded after 2 reminders (letter: 17/37, 45.9%; postcard: 20/37, 54.1%).

### Emotional Distress and Psychological Support

Table 2 presents results pertaining to emotional distress and psychological support. Current distress was commonly reported, and the majority of parents reported past experience of emotional distress. Of those parents who had sought help for their distress, the majority had received help. Help for emotional distress had been received from a variety of health professionals, with the majority receiving support from a psychologist, therapist, or physician. For those currently experiencing emotional distress, internet-administered psychological support, with guidance from a psychologist, was deemed appropriate by the majority (see Table 2). Furthermore, the majority parents (regardless of current emotional distress) reported that they would definitely or maybe accept internet-administered psychological support if offered.

### Past Experience of Research Participation and Attitudes Toward Research

Almost half of the parents had previously participated in research, and the majority responded that they held either very high or somewhat high trust in research (Table 3).

### Attitudes Toward Trial Procedures

Table 4 presents results regarding attitudes toward trial procedures. For receiving initial study information, the largest number of parents considered a postal letter as acceptable. In addition, the majority responded they would find presentation of further study information on a study website via text or video acceptable. While a researcher was most widely considered as an acceptable sender of study invitations, other professionals, including psychologists and nurses, were also considered acceptable by some parents (see Table 4). Furthermore, just under one-third of parents responded that they would find it acceptable to receive a study invitation from a parent of a child previously treated for cancer.

The majority of parents responded that they would either accept, or maybe accept, participation in a hypothetical RCT of an internet-administered psychological intervention utilizing a waiting list control condition. Little difference in the preference was found regarding an acceptable length of waiting list time; however, a slightly higher acceptance was reported for a waiting list length of 1-2 months (see Table 4). Moreover, the majority responded that they would accept, or maybe accept, participation in a hypothesized alternative active treatment and participant-preference trial.

**Table 2:** Emotional distress and preferences for psychological support (n=112).

Emotional distress and preferences			Value, n (%)
**Emotional distress**			
	**Current self-reported emotional distress**		
		Yes	41 (36.6)
		No	70 (62.5)
		Missing	1 (0.9)
	**Past emotional distress**		
		Yes	80 (71.4)
		No	32 (28.6)
**Psychological support**			
	**Sought help for emotional distress**		
		Yes	48 (42.8)
		No	33 (29.5)
		Not applicable (no past or current emotional distress)	31 (27.7)
	**Help received for emotional distress (of those who sought help)^a^**		
		Yes	39 (81.2)
		No	8 (16.7)
		Missing	1 (2.1)
	**Type of help received for emotional distress^a,b,c^**		
		Psychologist	14 (29.2)
		Therapist	13 (27.1)
		Physician	12 (25.0)
		Counselor	7 (14.6)
		Health care center	6 (12.5)
		Church	2 (4.2)
		Stress management self-help program	1 (2.1)
		Mindfulness Exercises	1 (2.1)
	**Preferences for psychological support for parents currently self-reporting distress^d^**		
		Other (unspecified)	13 (31.7)
		Internet-administered psychological treatment with support from a psychologist	11 (26.8)
		Internet-administered psychological treatment and to see a psychologist in person	11 (26.8)
		Internet-administered psychological treatment without support from a psychologist	3 (7.3)
		See a psychologist in person	2 (5.0)
		Missing	1 (2.4)
	**Accept internet-administered psychological support if offered**		
		Yes	47 (42.0)
		Maybe	36 (32.1)
		No	28 (25.0)
		Missing	1 (0.9)

^a^n=48.

^b^Multiple responses possible.

^c^Open-ended question.

^d^n=41.

**Table 3:** Past experience of research participation and attitudes toward research (n=112).

Experience with and attitude toward research		Value, n (%)
**Past experience of research participation**		
	No	56 (50.0)
	Yes	55 (49.1)
	Missing	1 (0.9)
**Trust in research**		
	Very high	56 (50.0)
	Somewhat high	51 (45.5)
	Moderate	5 (4.5)

**Table 4:** Attitudes concerning trial procedures (n=112).

Attitude			Value, n (%)	
**Receipt of initial study information^a^**				
	Postal letter		86 (76.8)	
	Meeting with a physician, psychologist, or nurse		34 (30.4)	
	Telephone		18 (16.1)	
	Short message service text message		13 (11.6)	
	Other		11 (9.8)	
**Presentation of study information on a study website^a^**				
	Text		81 (72.3)	
	Video		66 (58.9)	
	Image(s)		43 (38.4)	
	Audio		23 (20.5)	
**Who should send study invitations^a^**				
	Researcher		84 (75.0)	
	Psychologist		44 (39.3)	
	Nurse previously met		43 (38.4)	
	Parent of a child treated for cancer		36 (32.1)	
	Psychologist previously met		32 (28.6)	
	Nurse		30 (26.8)	
	Another option, not specified		11 (9.8)	
**Acceptability of controlled trial procedures**				
	**Waiting list randomized controlled trial**			
		Yes		53 (47.3)
		Maybe		41 (36.6)
		No		18 (16.1)
	**Length of waiting list**			
		1-2 months		20 (17.8)
		3-4 months		15 (13.4)
		5-6 months		17 (15.2)
		>6 months		18 (16.1)
		Other		11 (9.8)
		Would decline participation		17 (15.2)
		Missing		14 (12.5)
	**Active treatment randomized controlled trial**			
		Yes		49 (43.7)
		Maybe		42 (37.5)
		No		18 (16.1)
		Missing		3 (2.7)
	**Patient-preference controlled trial**			
		Yes		59 (52.7)
		Maybe		34 (30.3)
		No		17 (15.2)
		Missing		2 (1.8)

^a^Participants could select multiple options.

## Discussion

### Principal Findings

This cross-sectional, Web-based survey examined attitudes and preferences toward and willingness to participate in a hypothetical trial of an internet-administered psychological intervention for parents of children treated for cancer. Furthermore, differences in the response rate between two modes of study invitation (standard letter vs professionally illustrated postcard) were examined. To the best of our knowledge, this is the first survey to examine attitudes and preferences toward the design of and participation in internet-administered psychological intervention trials within the population. Summarizing the main findings, an overall 32.0% (112/350) response rate was yielded, yet no difference was found in the response rate between modes of study invitation. Self-reported current and past emotional distress was common. Internet-administered psychological support, under guidance of a psychologist, was deemed acceptable by the majority of those parents currently reporting emotional distress and of the surveyed population as a whole. Examination of acceptable recruitment methods indicated the most acceptable method of study invitation would be via postal letter, with full study information presented on a study website via text or video and delivered by a researcher. On the whole, parents responded that they would either accept, or maybe accept, participation in each presented hypothetical trial design (waiting list control, alternative active treatment, patient-preference), with no overall indication of a preferred trial design.

It is interesting to note that no association between the mode of invitation and the response rate was detected. As such, this suggests professional illustration may not increase study response rates. This finding is in contrast to research indicating that professionally designed invitation packs can improve recruitment rates [36]. Still, other studies on the optimization of patient information sheets via professional design and user-testing have failed to yield larger response rates compared with providing standard study information [37,38]. Given these findings, the time and financial resources required to engage a professional design service may not be warranted. A note of caution is, however, due here as the main design modification was the use of professional illustration and presentation via a postcard versus standard study invitation letter. Future research may be required to examine whether additional design modifications (eg, language, structure, and length) may impact response rates.

Another noteworthy finding was that parents showed an overall preference for receiving initial study invitations via the post. While postal recruitment from disease registers is a common recruitment strategy [39], evidence suggests that providing telephone reminders enhances recruitment rates [40]. Only 16.1% (18/112) of parents considered study invitation via the telephone as acceptable. However, this survey specifically examined the acceptability of telephone contact regarding the receipt of initial study invitation, rather than preferred ways of receiving study invitation reminders. Further work may aim to examine what methods of sending study reminders would be considered acceptable. Moreover, it is interesting to note that the majority of parents responded receiving full study information on Web in text and video format would be perceived as acceptable. Indeed, research suggests the provision of trial information via video, supplementing the provision of written information, may improve participation rates [40].

Interestingly, similar percentages of parents responded they would agree, or maybe agree, to participate in trials using waiting list control, alternative active treatment, and patient-preference designs. Furthermore, little difference was found concerning acceptable waiting times for a hypothetical waiting list RCT. As such, the majority responded they would be willing to participate in hypothetical trial designs utilizing randomization procedures. This finding is in contrast to research reporting that a fear of randomization is a common reason for declining trial participation [41]. One potential explanation for this finding may be the presentation of an informational video to participants explaining randomization and different designs presented in the survey. Indeed, previous research has demonstrated that clinical trial educational videos can reduce barriers to trial participation and increase preparedness to consider clinical trial enrollment [42]. Furthermore, almost half of the parents responded that they had previously participated in research, and almost all reported high to moderately high levels of trust in research. Again, this finding contrasts with previous research, whereby fear and mistrust of clinical trial research has been reported [43]. Given many parents had participated in past research, the surveyed population may already have increased levels of knowledge regarding trial design, resulting in more positive attitudes toward trial participation.

In line with previous research [9,11], a susbtantial proportion of parents responded that they had experience of past and current emotional distress. This finding further supports research demonstrating parents experience a need for psychological support from the end of cancer treatment, persisting into the long term [12]. Although in contrast to previous research [12-14], the majority who had sought support for their distress had received support from a health care professional. Despite this, it is important to bear in mind that the survey did not examine the type, quality, or acceptability of psychological support received by parents. In addition, survey results indicate that internet-administered psychological interventions, supported by a psychologist, potentially represent an acceptable type of support for parents of children previously treated for cancer. These findings are in line with wider research indicating generally high levels of acceptability for the delivery of internet-administered psychological interventions [21]. However, future research is required to examine the acceptability of the internet-administered psychological intervention from the perspective of actual intervention users.

### Limitations

This study has several limitations. First, willingness to participate in a hypothetical trial of an internet-administered psychological intervention may not predict actual trial enrollment [44]. Even though the majority of respondents demonstrated a willingness to participate in a hypothetical trial, findings may not represent the decision made if parents were offered participation in a “real-life” trial. A related limitation pertains to the examination of response rates. The study was a brief Web-based survey; however, trial participation requires time and commitment [39]. Therefore, response rates obtained in this Web-based survey may not reflect actual trial response rates. In addition, there was no formal measurement of emotional distress utilizing a standardized and validated self-report or clinician-administered measurement of distress. As a consequence, it is difficult to determine whether parents were experiencing levels of emotional distress suitable for psychological support. Thus, findings may not be generalizable to parents experiencing clinical levels of emotional distress appropriate for formal psychological support. Still, percentages of parents responding that they had experienced either past of or current emotional distress are in line with earlier studies examining clinically relevant levels of psychological distress in the population [11]. An additional limitation is the small survey sample size, resulting from a 32% response rate. This is especially important considering parents who did not respond may have different attitudes and preferences toward trial design and conduct. Indeed, previous work suggests a category of nonresponders to research labeled “prior decliners” who have an established and unmodifiable position of declining participation in research [45]. Importantly, the majority of parents who did respond had previously participated in research, and therefore, surveyed sample may represent parents with higher levels of acceptability concerning research.

Another limitation pertains to only examining the acceptability of more traditional clinical trial recruitment strategies, for example, postal recruitment and clinician referral. However, evidence suggests Web-based recruitment strategies are effective for internet-administered intervention trials [28,46] and future studies may wish to examine attitudes and preferences toward Web-based recruitment strategies. The adoption of postal, rather than Web-based, recruitment strategies may also have reduced the survey participation rate. A further limitation concerns the cross-sectional survey design adopted. Adopting a mixed-methods approach may have facilitated a more thorough exploration of research questions and aided interpretation of survey findings. Finally, the order of response alternatives may have resulted in primacy effects.

Notwithstanding these limitations, this is to our knowledge the first study to examine attitudes toward clinical trials, preferences regarding study design, and willingness to receive internet-administered psychological support among parents of children previously treated for cancer. The results from this study will have considerable implications for the design of a planned feasibility study [47], such as initial study invitations being sent via postal letters by the research team, full study information being presented on Web via both text and video, and the provision of support from a psychologist.

### Conclusions

Clinical trial conduct is time and resource intensive. While research has been performed to examine attitudes and preferences toward clinical trial design and participation [33,48,49], little is known about attitudes and preferences toward trial design and participation in this study population. What also remains unclear is the acceptability of internet-administered psychological interventions from the perspective of parents of children previously treated for cancer. This study builds upon prior research to develop an ICBT intervention for the population and preliminary investigation of the acceptability of planned study procedures [10,23]. Survey findings have further enhanced our understanding of the acceptability of internet-administered approaches for the population, alongside an appreciation of potentially acceptable study procedures and design. Results will inform the design of a feasibility study of an ICBT intervention for parents of children previously treated for cancer [47], to further examine methodological, procedural, and clinical uncertainties [25,50].

## Appendix

Multimedia Appendix 1 Postcard and letter invitation text (English translation).

Multimedia Appendix 2 Full study information (English translation).

Multimedia Appendix 3 Informed consent (English translation).

Multimedia Appendix 4 Informational video (Swedish).

Multimedia Appendix 5 Informational video (English transcript).

Multimedia Appendix 6 Informational video PowerPoint slide (English translation).

Multimedia Appendix 7 Web-based survey (English translation).

## Abbreviations

ICBT: internet-administered cognitive behavioral therapy
MRC: Medical Research Council
RCT: randomized controlled trial
