# Supplementary material for: Attitudes and Preferences Toward a Hypothetical Trial of an Internet-Administered Psychological Intervention for Parents of Children Treated for Cancer: Web-Based Survey
Source: JMIR Ment Health. 2018 Dec 18;5(4):e10085. doi: 10.2196/10085 (PMC6318150; doi:10.2196/10085)
Supplement: Multimedia Appendix 1 [file mental_v5i4e10085_app1.pdf]

Multimedia Appendix 1: Postcard and letter invitation text (English translation)

Hi!
We are a group of researchers at Uppsala University who have developed an Internet-based psychological self-help programme for parents together with the parents of children diagnosed with cancer. If you would like to be involved in looking into how researching the programme could best be done and have 20 minutes to spare, click on the link www.u-care.se/parentscan.

Your study code is: xxxxxx.

Thank you for your help!

Best regards,

Professor Louise von Essen, responsible researcher

Contact details:
Professor Louise von Essen
Phone: +46(0)704250714
E-mail: louise-von.essen@pubcare.uu.se
Department of Public Health and Caring Sciences
Uppsala University
