# Supplementary material for: Attitudes and Preferences Toward a Hypothetical Trial of an Internet-Administered Psychological Intervention for Parents of Children Treated for Cancer: Web-Based Survey
Source: JMIR Ment Health. 2018 Dec 18;5(4):e10085. doi: 10.2196/10085 (PMC6318150; doi:10.2196/10085)
Supplement: Multimedia Appendix 2 [file mental_v5i4e10085_app2.pdf]

Multimedia Appendix 2: Full study information (English translation)

Returning to everyday life after a child has been treated for cancer can present challenges for the whole family. It is not unusual for parents to experience emotional distress after their child has completed cancer treatment.

In our research team, we are developing an Internet-based psychological self-help programme for parents who are experiencing emotional distress after their child has successfully completed treatment for cancer. To find out whether the programme helps parents, we intend to carry out a study. Before we start the study, we want to know how parents of children treated for cancer think the study should be carried out. We have obtained your personal data from the Swedish Childhood Cancer Register and Statens personadressregister (SPAR), Sweden's address register for residents, and wonder if you would like to help us to identify how best to carry out this study. If you want to, you can first watch a short video about scientific studies before answering some questions.

Your participation will take approximately 20 minutes. We have not identified any risks associated with participation in this survey. You might experience it ~~as~~ beneficial to have the opportunity to influence future research and contribute to the development of an Internet-based self-help programme for parents of children treated for cancer who are in need of psychological support.

If you are interested in participating, please continue reading under the tab "Informed Consent"!
