# Supplementary material for: Attitudes and Preferences Toward a Hypothetical Trial of an Internet-Administered Psychological Intervention for Parents of Children Treated for Cancer: Web-Based Survey
Source: JMIR Ment Health. 2018 Dec 18;5(4):e10085. doi: 10.2196/10085 (PMC6318150; doi:10.2196/10085)
Supplement: Multimedia Appendix 3 [file mental_v5i4e10085_app3.pdf]

Multimedia Appendix 3: Informed consent (English translation)

All data concerning your participation in this study will be processed in ways that prevent un-authorized persons from knowing what answers you have provided. Processing your personal data will be governed by Sweden's Data Protection Act (PUL, SFS 1998:204), where Uppsala University has responsibility for your personal data under this Act. No personal data will be disclosed to any third party. The results of the study will be presented at group level in scientific journals and it will not be possible to identify any individual. Participation is voluntary and you can cease your participation at any time without telling us why and without any consequences, even after you have begun participating.

Below, you can consent to participate in the study. If you have any questions, you are welcome to contact PhD student xxxx xxxx, e-mail: xxx.xxx@pubcare.uu.se, Department of Public Health and Caring Sciences, Uppsala University, phone number +46(0)xx-xxx xx xx.

☐ I hereby consent to participate in the study and consent to my personal data being processed in the manner described above.

Name:	
Address:	
Phone no.:	
Study code:	

The responsible authority for the research is Uppsala University
Contact details for the responsible researcher:
Professor Louise von Essen
Phone: +46(0)18-4713484, +46(0)70 4250714, e-mail: louise-von.essen@pubcare.uu.se
Department of Public Health and Caring Sciences
Uppsala University
