# Supplementary material for: Attitudes and Preferences Toward a Hypothetical Trial of an Internet-Administered Psychological Intervention for Parents of Children Treated for Cancer: Web-Based Survey
Source: JMIR Ment Health. 2018 Dec 18;5(4):e10085. doi: 10.2196/10085 (PMC6318150; doi:10.2196/10085)
Supplement: Multimedia Appendix 5 [file mental_v5i4e10085_app5.pdf]

Multimedia Appendix 5: Informational video (English transcript)

Thank you for your interest in participating in our research!
In this short video, I will briefly describe a little bit about our activities and how a research study is carried out.

My name is [researcher name] and I work as a research assistant at Uppsala University. My colleagues and I are working on developing an Internet-based psychological treatment for parents experiencing emotional distress after their child has successfully completed cancer treatment.
In a study, we aim to investigate whether the treatment is helpful and would like help from you and other parents in planning how we should carry out this study. With help from you and other parents, we hope to be able to improve our activities.

Being the parent of a child diagnosed with cancer is very stressful, but some parents end up feeling a bit worse than others, experiencing anxiety, depression or post-traumatic stress for example. Parents can experience these problems during their child's treatment or even a long time after the treatment has ended. We know that many parents in this situation who want help to deal with emotional distress do not get access to this kind of help. With the aid of the Internet, it's possible to offer psychological treatment to a greater number of people than if the treatment is provided face-to-face. It has been shown that Internet-based treatment can be just as effective as meeting with a psychologist in person.

Internet-based psychological treatment means using a PC, tablet or smartphone to access texts, videos, audio files and exercises for example. There is also the option of contacting a psychologist via messaging, phone call or video call. You can also contact other parents in similar situations.

We have shown that Internet-based psychological treatment can reduce anxiety, depression or post-traumatic stress in parents whose children were recently diagnosed with cancer. Because we know that for many parents it's difficult to find time for oneself during the period when their child is undergoing cancer treatment, we are now developing a psychological treatment for parents whose child has completed cancer treatment. We know that many of these parents feel bad but are not getting help to deal with their problems.

Before we offer the treatment to the parents, we want to evaluate the effect of the treatment in a research study. We want to develop a treatment that helps parents and a research study that parents want to participate in. It's also important that those included continue their participation to the end of the study. A common way to evaluate the effects of a treatment is to conduct a research study where the participants are randomly selected to receive treatment immediately or at a later date. This kind of study can be illustrated in the following way:

Here we have a group of parents who are experiencing problems in the form of anxiety, depression or post-traumatic stress resulting from their child's cancer diagnosis.
After the parents have consented to participate in the study and reported how they feel, they are randomly selected to receive treatment either immediately or later.
After those who have been selected for immediate access to the treatment have completed the treatment, they are compared to those who have not yet received the treatment.
If those who received the treatment report a reduction in emotional distress compared with those who have not received the treatment, the treatment has had a positive impact. After this has been investigated, the group waiting for treatment gets access to the treatment. Another common way of evaluating the efficacy of a treatment is to conduct a research study where the participants are randomly selected to receive one of two therapies. This kind of study can be illustrated in the following way:
Here we have a group of parents who are experiencing problems in the form of anxiety, depression or post-traumatic stress resulting from their child's cancer diagnosis.
After the parents have consented to participate in the study and reported how they feel, they are randomly selected to receive treatment A or treatment B.

After the participants have completed their treatment, the researchers compare how those who were given access to treatment A feel compared with those who were given access to treatment B.
If one of the groups reports a reduction in their problems compared with the other group, the treatment that the first group received has had a positive effect.

If those invited to participate in a study where two treatments are compared with each other have a strong preference for one of the two treatments, this can mean that few want to participate in the study, or that many don’t complete the study. This leads to difficulties in determining whether one of the treatments is more effective than the other.
There is the option of allowing people to choose the treatment they want access to, but it then becomes a bit more difficult to determine which of the treatments was the most effective.

We will now ask you to answer a number of questions about how you feel, what type of psychological treatment you would prefer, and how you think a research study in this area should be carried out.

Many thanks for participating in our research!

You can now continue to the survey.
