# Supplementary material for: Attitudes and Preferences Toward a Hypothetical Trial of an Internet-Administered Psychological Intervention for Parents of Children Treated for Cancer: Web-Based Survey
Source: JMIR Ment Health. 2018 Dec 18;5(4):e10085. doi: 10.2196/10085 (PMC6318150; doi:10.2196/10085)
Supplement: Multimedia Appendix 6 [file mental_v5i4e10085_app6.pptx]

## Slide 1
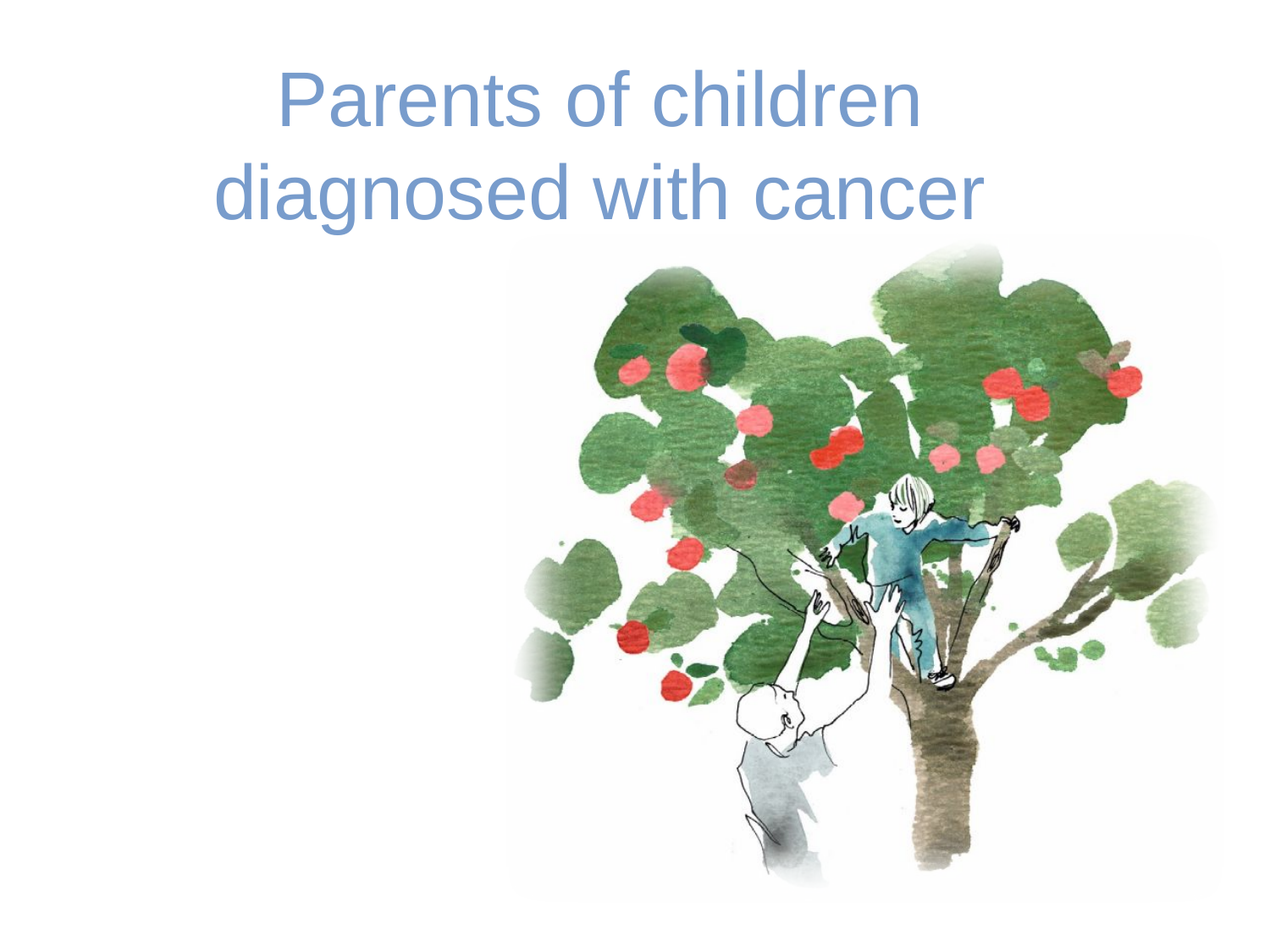

# Parents of children diagnosed with cancer

## Slide 2
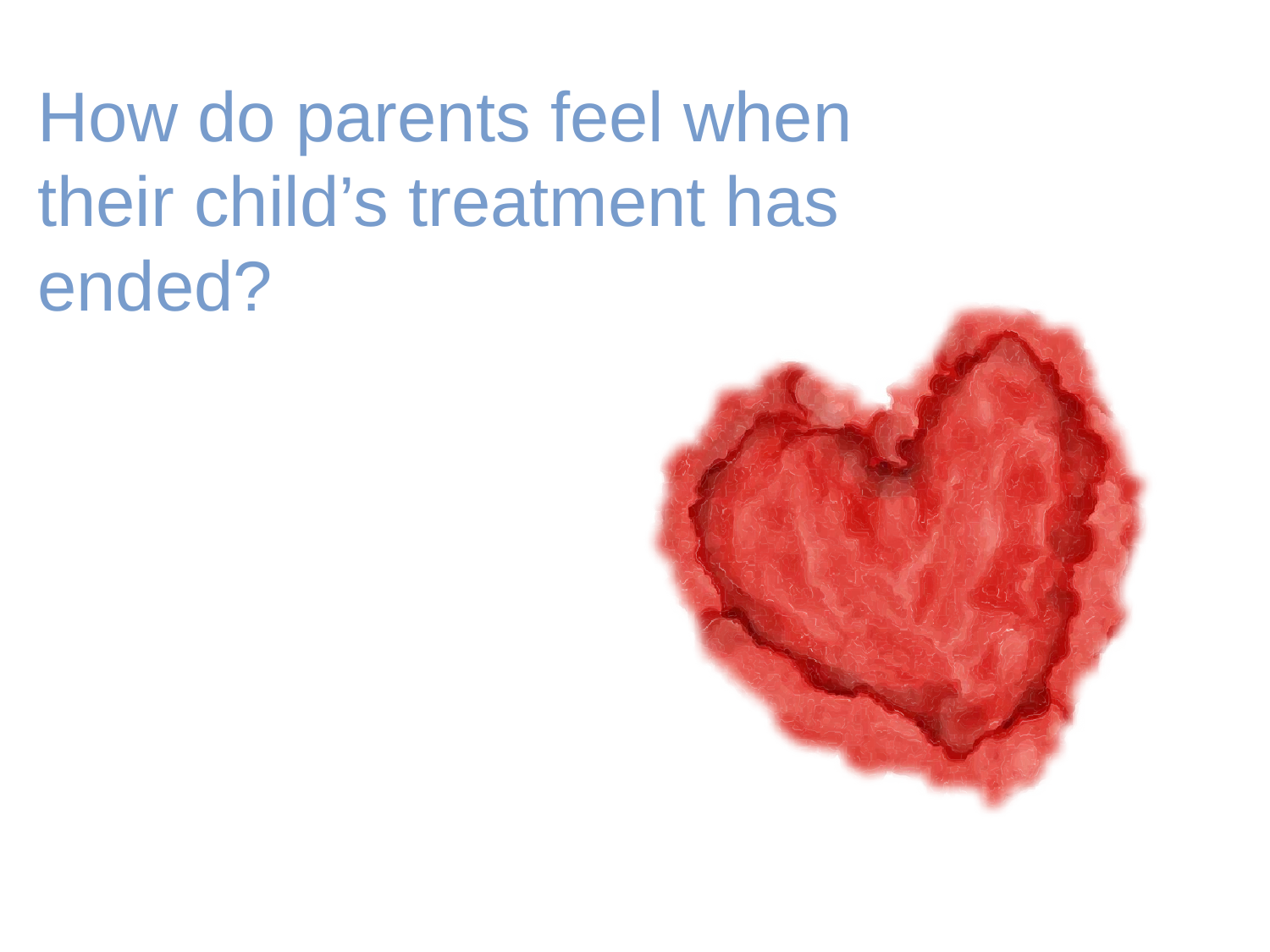

# How do parents feel when their child’s treatment has ended?

## Slide 3
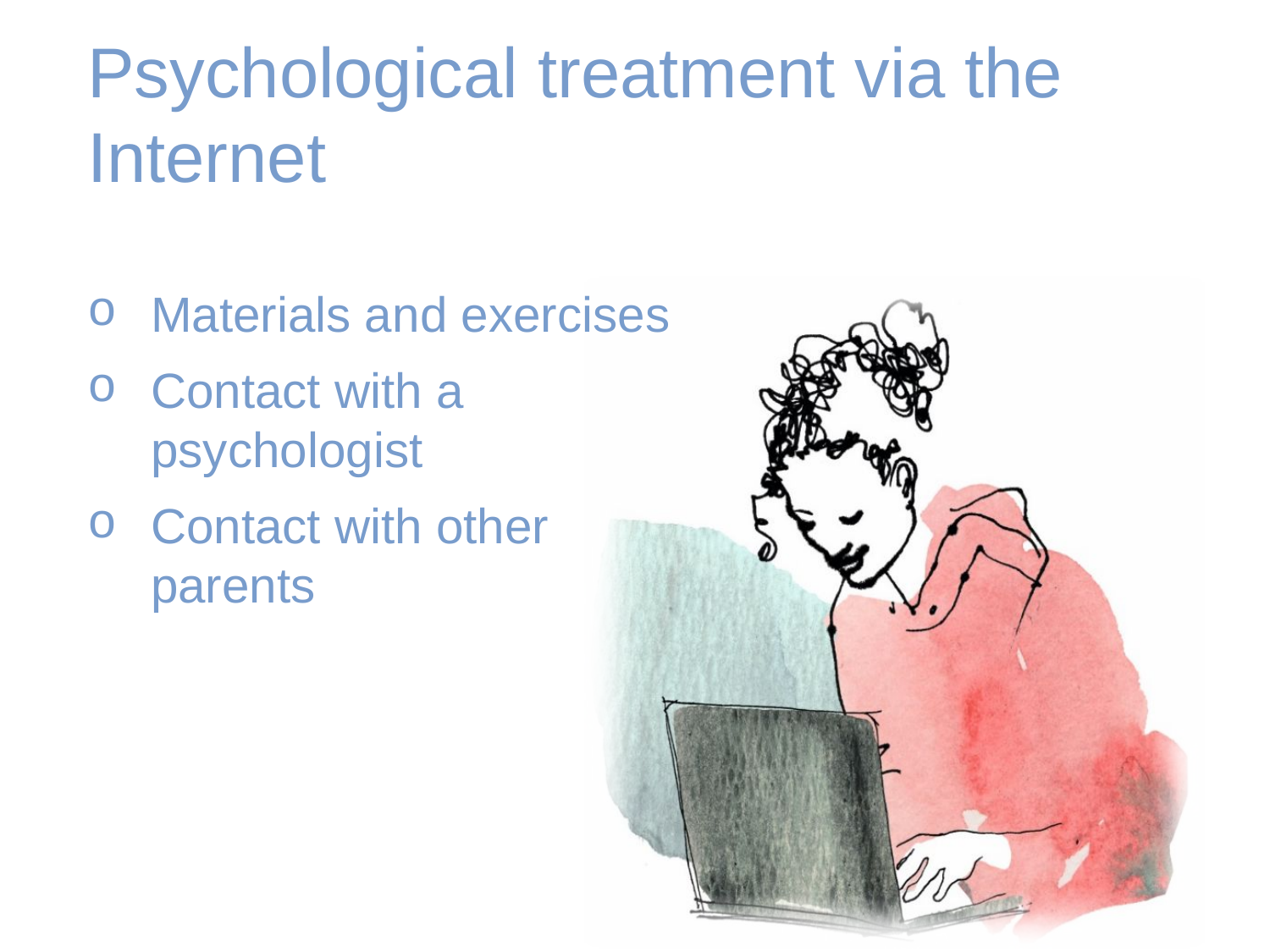

# Psychological treatment via the Internet
Materials and exercises
Contact with a psychologist
Contact with other parents

## Slide 4
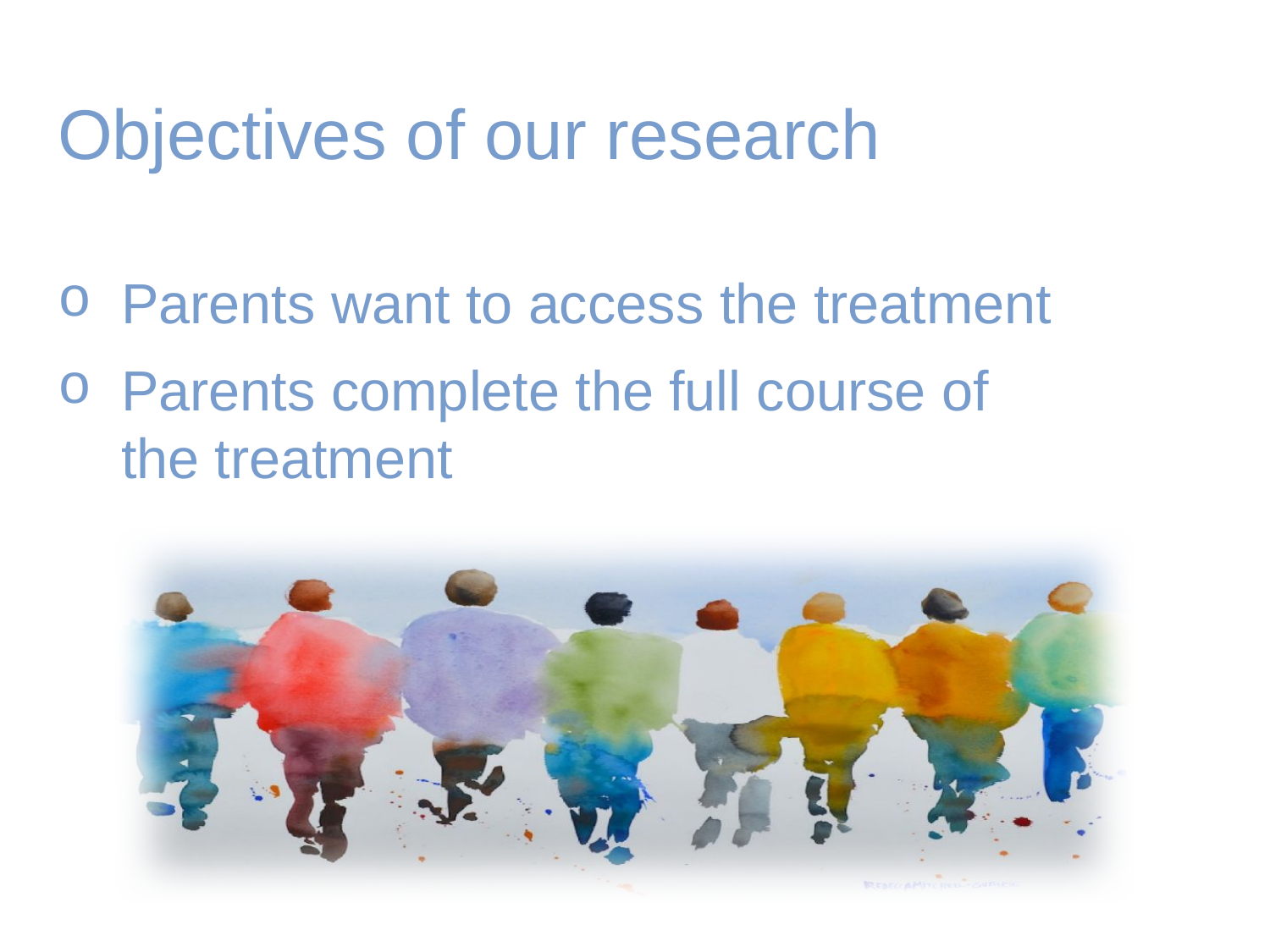

# Objectives of our research
Parents want to access the treatment
Parents complete the full course of the treatment

## Slide 5
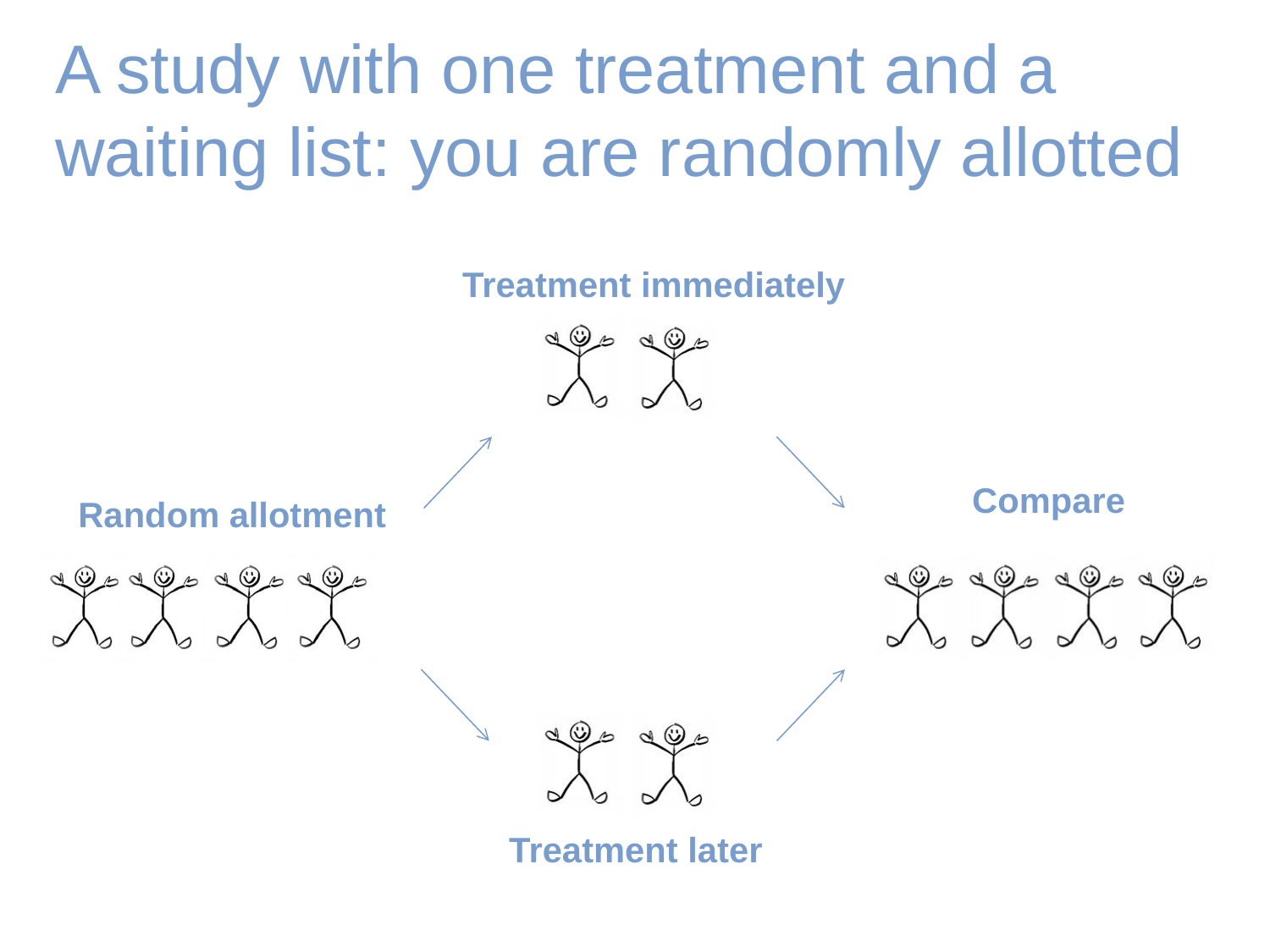

# A study with one treatment and a waiting list: you are randomly allotted
Treatment immediately
Compare
Random allotment
Treatment later

## Slide 6
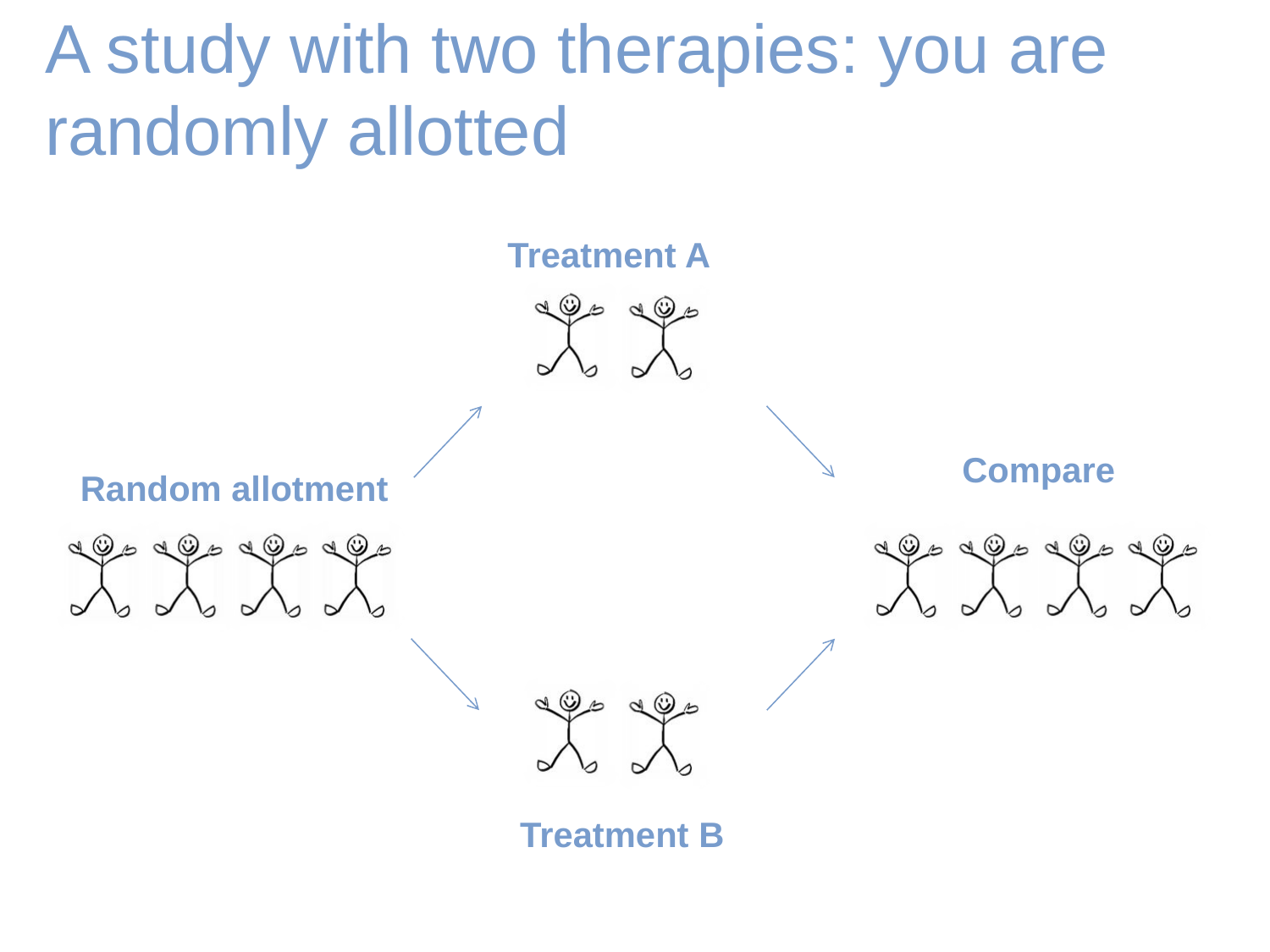

# A study with two therapies: you are randomly allotted
Treatment A
Compare
Random allotment
Treatment B

## Slide 7
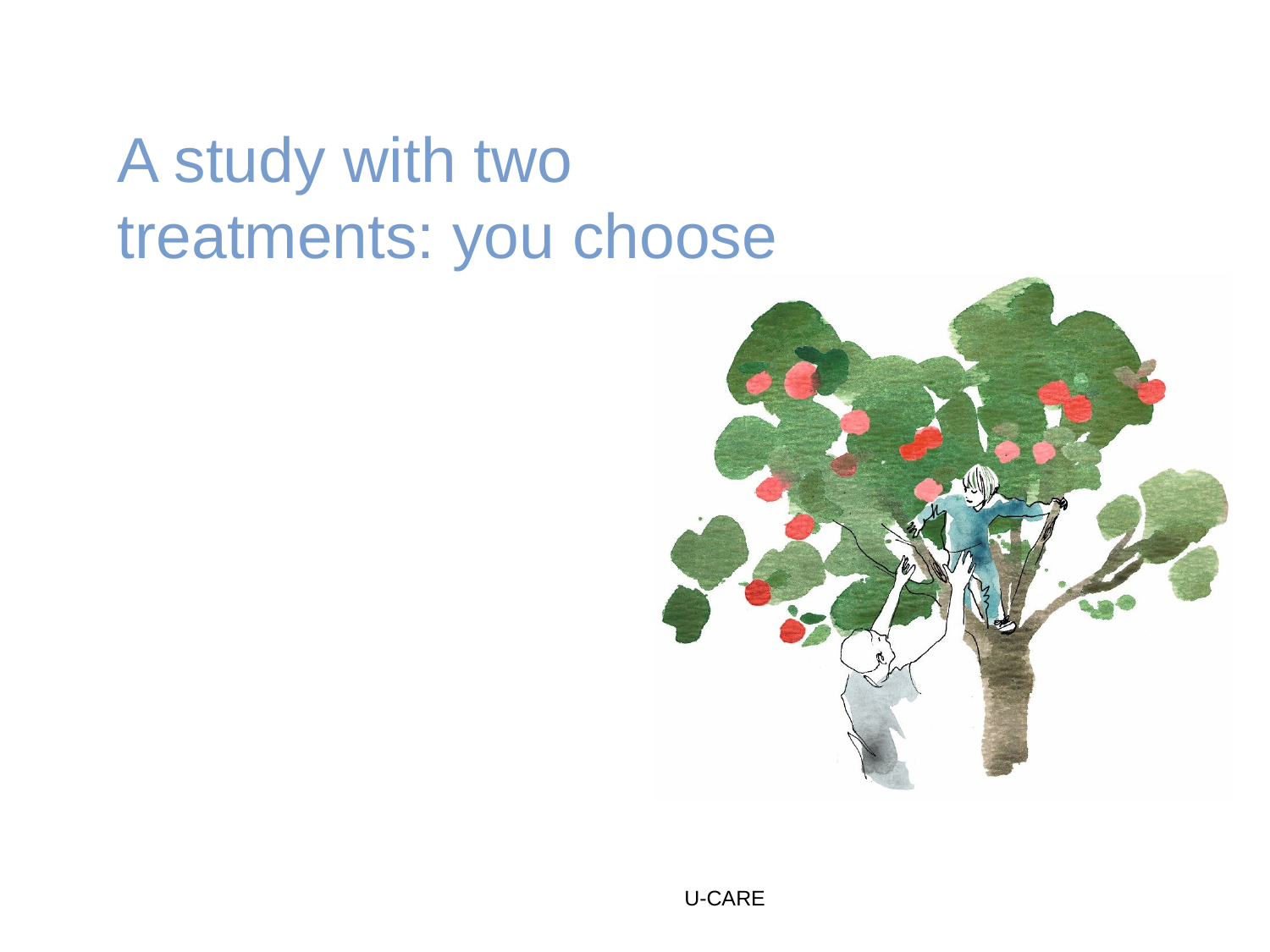

# A study with two treatments: you choose
U-CARE

## Slide 8
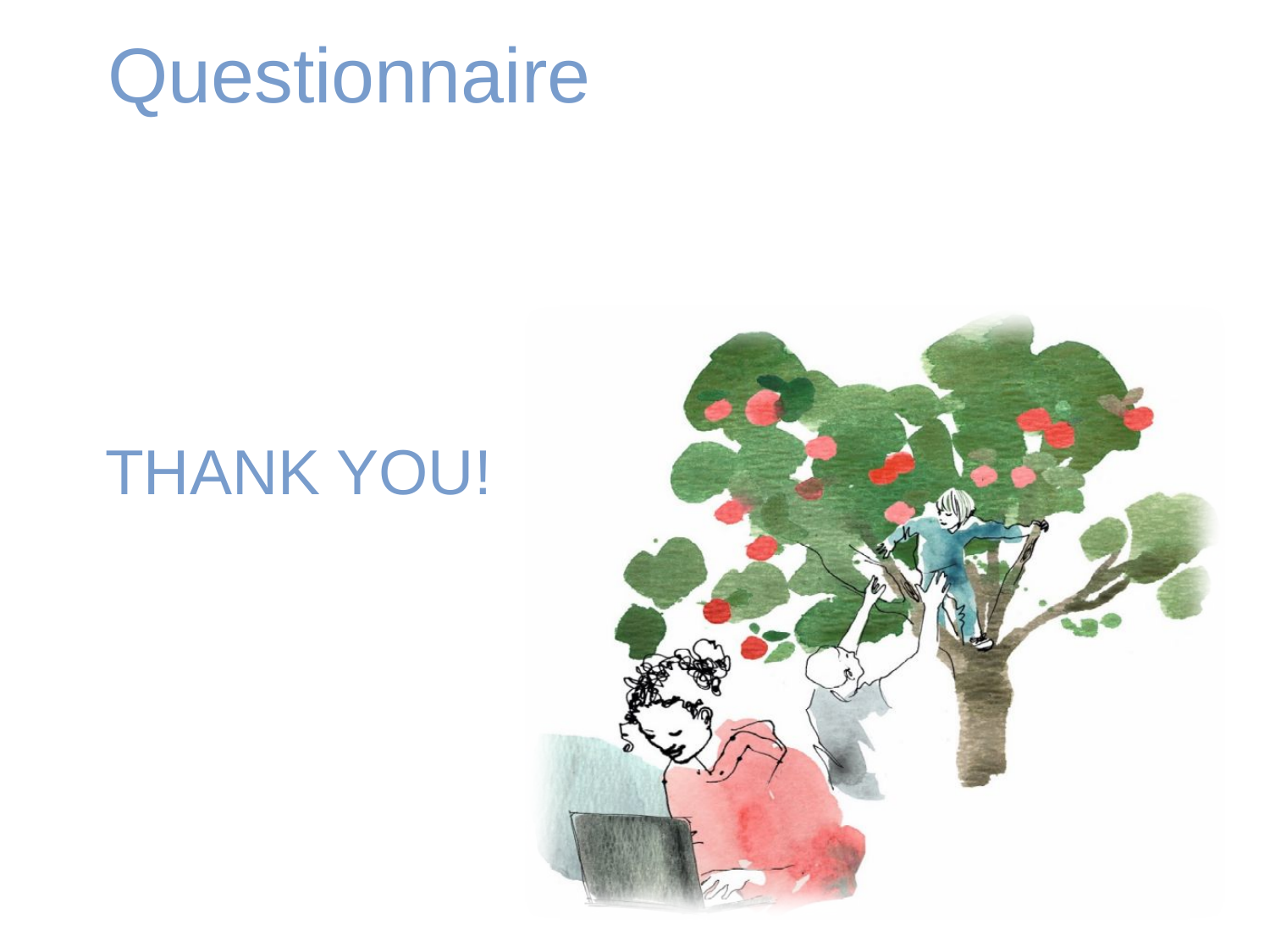

# Questionnaire
	THANK YOU!
