# Supplementary material for: Attitudes and Preferences Toward a Hypothetical Trial of an Internet-Administered Psychological Intervention for Parents of Children Treated for Cancer: Web-Based Survey
Source: JMIR Ment Health. 2018 Dec 18;5(4):e10085. doi: 10.2196/10085 (PMC6318150; doi:10.2196/10085)
Supplement: Multimedia Appendix 7 [file mental_v5i4e10085_app7.pdf]

Multimedia Appendix 7: Online survey (English translation)

1. Gender

☐ Female

☐ Male

☐ Do not want to answer this question.

2. Year of birth: 19 __ __

3. When did the treatment of your child's cancer disease end? If your child has had more than one cancer disease/ relapse, please state the treatment end date for the latest cancer disease?
MM: __ __ YY: __ __

4. Do you currently experience emotional distress related to your child's cancer disease?

☐ Yes

☐ No

Feel free to comment on your response:

5. Have you previously experienced emotional distress, after treatment for your child's cancer disease ended, related to your child's cancer disease?

☐ Yes

☐ No

Feel free to comment on your response:

6. If you responded yes to question 4 and/or 5, did you seek help?

☐ Yes

☐ No

Feel free to comment on your response:

7. If you responded yes to question 6, did you receive help?

☐ Yes

☐ No

If you received help, what kind of help was it?

Feel free to comment on your response:

8. If you currently experience emotional distress due to your child's cancer disease - what kind of help would you prefer?

☐ Internet-administered psychological treatment without support from a psychologist

☐ Internet-administered psychological treatment with support from a psychologist

☐ Internet-administered psychological treatment and to see a psychologist in person

☐ See a psychologist in person

☐ Other

Feel free to comment on your response:

Feel free to describe what kind of help you would prefer:

9. If you were offered an Internet-administered psychological treatment, would you accept it?

☐ Yes

☐ No

☐ Maybe

Feel free to comment on your response:

10. Do you (not your child) have any previous experience of participation in a research study? For example: drug trials, surveys, interviews.

☐ Yes

☐ No

Feel free to comment on your response:

11. To what extent do you generally trust research?

☐ Very high trust

☐ Somewhat high trust

☐ Moderate trust

☐ Somewhat low trust

☐ Very low trust

Feel free to comment on your response:

12. If the opportunity arose, would you like to participate in a research study where participants would gain access to an Internet-administered psychological treatment with support from a psychologist?

☐ Yes

☐ No

☐ Maybe

Feel free to comment on your response:

13. How would you like to receive the initial information about a research study? (You can choose multiple options)

☐ Letter

☐ Personal meeting with a doctor, psychologist or nurse

☐ Text message

☐ Phone call

☐ Other

Feel free to comment on your response:

14. If you were given additional information about a research study through a website, how would you like this information to be presented? (You can choose multiple options)

☐ Image

☐ Video

☐ Audio

☐ Text

☐ Other

Feel free to comment on your response:

15. From whom would you want to receive an invitation to participate in a research study? (You can choose multiple options.)

☐ A researcher

☐ A parent of a child who has been treated for cancer.

☐ A psychologist who you have previously met

☐ A psychologist

☐ A nurse who you who you have previously met

☐ A nurse

☐ Other

Feel free to comment on your response:

16. Would you agree to participate in a research study on condition that after agreeing to participate you would be randomised either to access an Internet-administered psychological treatment immediately, or to access the same treatment after a waiting time?

☐ Yes

☐ No

☐ Maybe

Feel free to comment on your response:

17. If you responded yes to question 16, how long would you consider it acceptable to be waiting before gaining access to treatment?

☐ 1-2 months

☐ 3-4 months

☐ 5-6 months

☐ More than 6 months

☐ Other

☐ I would have declined participation if I had to wait.

Feel free to comment on your response:

18. Would you agree to participate in a research study on condition that after you had agreed to take part you would be randomised either to access an Internet-administered psychological treatment or another type of treatment?

☐ Yes

☐ No

☐ Maybe

Feel free to comment on your response:

19. Would you agree to participate in a research study on condition that after you had agreed to take part you would be able to decide which of two treatments you wanted to gain access to?

☐ Yes

☐ No

☐ Maybe

Feel free to comment on your response:

20. Is there anything else you would like us researchers to consider in order to increase peoples' interest in participating in research studies?
